# Helminth–virus interactions: determinants of coinfection outcomes

**DOI:** 10.1080/19490976.2021.1961202

**Published:** 2021-08-24

**Authors:** Pritesh Desai, Michael S. Diamond, Larissa B. Thackray

**Affiliations:** a Department of Medicine, Washington University School of Medicine, Saint Louis, MO, United States; b Pathology & Immunology, Washington University School of Medicine, Saint Louis, MO, United States; c Molecular Microbiology, Washington University School of Medicine, Saint Louis, MO, United States; d The Andrew M. And Jane M. Bursky Center for Human Immunology and Immunotherapy Programs, Washington University School of Medicine, Saint Louis, MO, United States

**Keywords:** Helminth, virus, coinfection, intestine, tropism, antiviral, immunity, type 2 response

## Abstract

Viral infections are often studied in model mammalian organisms under specific pathogen-free conditions. However, in nature, coinfections are common, and infection with one organism can alter host susceptibility to infection with another. Helminth parasites share a long coevolutionary history with mammalian hosts and have shaped host physiology, metabolism, immunity, and the composition of the microbiome. Published studies suggest that helminth infection can either be beneficial or detrimental during viral infection. Here, we discuss coinfection studies in mouse models and use them to define key determinants that impact outcomes, including the type of antiviral immunity, the tissue tropism of both the helminth and the virus, and the timing of viral infection in relation to the helminth lifecycle. We also explore the current mechanistic understanding of how helminth-virus coinfection impacts host immunity and viral pathogenesis. While much attention has been placed on the impact of the gut bacterial microbiome on immunity to infection, we suggest that enteric helminths, as a part of the eukaryotic macrobiome, also represent an important modulator of disease pathogenesis and severity following virus infection.

## Introduction

Almost all mammalian species are inhabited by one or more helminths.^1^ The earliest record of helminth infection in humans is found in mummified individuals dating back thousands of years as well as in ancient medical writings.^2,3^ Earlier, helminth infections were widespread in the tropics and subtropics, and it is only in the last century that many industrialized nations have become largely helminth-free due to access to clean water and improved sanitation and hygiene practices.^4^ Since helminths share a long coevolutionary history with humans, their eradication is thought to have caused changes to host physiology, metabolism, and immunity, and explained a rise in allergic and inflammatory diseases.^5^ Notwithstanding these points, a quarter of the world’s population remains infected by helminths, making helminth coinfection a major modulator of host susceptibility to infectious diseases.^6,7^ Indeed, given the substantial geographical overlap between helminth-endemic areas and the prevalence of prominent infectious diseases including malaria, tuberculosis, human immunodeficiency virus (HIV), and hepatitis C virus, it is possible that helminths alter host susceptibility to many pathogenic microorganisms.^8–10^

Helminths that commonly infect humans establish patency in the gastrointestinal tract (GI) tract and are collectively known as GI helminths or enteric helminths.^6,11^ GI helminths include *Ascaris lumbricoides (A. lumbricoides), Trichuris trichiura (T. trichiura), Necator americanus (N. americanus)*, and *Ancylostoma duodenale (A. duodenale)*, which collectively infect more than two billion people worldwide.^6^ Some of these helminths enter their host via skin penetration in the form of infective larvae, whereas others enter via the fecal-oral route in the form of embryonated eggs or infective larvae usually through consumption of contaminated water. Inside the host, GI helminths undergo developmental molts to generate mature adult larvae that establish infection in the GI tract. Some GI helminths also traverse through other organs such as the lungs before establishing patency in the GI tract.^6^ In addition to GI helminths, the parasitic flatworm *Schistosoma* is also a major public health concern as it infects over 250 million individuals worldwide every year, mostly in the tropical regions.^11^ *Schistosoma spp*. have a complex life cycle where they invade multiple tissues including the lungs and the liver, before establishing chronicity in the portal vein and depositing their eggs into the lumen of the GI tract.^12^ Intestinal helminth infections are rarely lethal, but often cause morbidity in immunocompromised individuals and children including intestinal bleeding, iron deficiency, and protein malnutrition.^6,13–15^ However, in most infected individuals, the worm burden is low with no signs of overt clinical disease.^6^ This is in part due to the adaptation of helminths to their mammalian hosts and their utilization of immune evasion strategies that enable them to persist with limited tissue damage.^16^ Moreover, the host develops tolerance as a strategy to prevent the adverse effects of helminth-mediated or immune-mediated tissue damage.^17^ Such commensal-like adaptation, although mutually beneficial to helminths and host could inform host responses to subsequent interactions with heterologous pathogens such as viruses.

Although considerable field studies have been performed to determine the effect of helminth infection status on susceptibility to heterologous pathogens including viruses, there is limited mechanistic insight as to how these interactions affect outcomes. In this review, we discuss different scenarios in which enteric helminth coinfections have been reported to be beneficial or detrimental during viral infections. Using coinfection studies in mouse models, we discuss known molecular mechanisms involved in helminth–virus interactions and propose three main determinants that influence this interaction. Finally, we raise outstanding questions in the field of helminth-virus coinfection that could lead to future studies.

## Beneficial outcomes

### Enteric helminths and respiratory viruses

Enteric helminth coinfections can be beneficial against diseases caused by respiratory viruses. While in some cases lung disease is directly caused by respiratory viruses, in other situations morbidity is associated with excess infiltration of immune cells in the lungs that compromises pulmonary mechanics and gas exchange.^18^ One mechanism by which enteric helminths confer benefits against disease pathogenesis caused by respiratory viruses is by mitigating lung immunopathology. Limiting the infiltration of immune cells to the lungs or changing the quality of the immune response can lessen pulmonary inflammation and improve survival. Using mouse models, the impact of helminth coinfection on some respiratory viruses has been examined. The murine helminth *Heligmosomoides polygyrus bakeri* (*H. polygyrus; previously called Nematospiroides dubius)* is widely utilized as a model of human GI hookworm infection. Early studies showed that coinfection of mice with *H. polygyrus* and influenza virus decreased the lung consolidation caused by influenza virus infection.^19,20^ Although the mechanisms underlying these changes were not fully addressed, the investigators speculated that this might be due to the immunosuppressive effect of *H. polygyrus*. Alternatively, the observed protection against pulmonary disease might be due to reduced infiltration of inflammatory immune cells into the lungs. Corroborating evidence for this latter explanation came from a study showing that enteric helminth infection modulates the trafficking patterns of immune cells such that their numbers increase in local lymphoid tissues (*e.g*., mesenteric lymph nodes) and decrease in peripheral lymphoid tissues.^21^ Consequently, activation of immune cells in peripheral lymphoid tissues and their migration to extraintestinal tissue compartments such as the lungs was reduced. This effect was also seen during coinfection of mice with the enteric helminth *Trichinella spiralis* (*T. spiralis*) and influenza virus, with reduced infiltration of neutrophils, natural killer (NK) cells, and T cells in the lungs of coinfected mice.^22^ In theory, altered trafficking of immune cells could also affect the accumulation of protective immune cells in the lungs and compromise antiviral immunity leading to increased viral burden. However, despite mounting suboptimal virus-specific CD8^+^ T cell response in the lungs and draining lymph nodes of coinfected mice,^21,23^ coinfection with *H. polygyrus* or *T. spiralis* resulted in only minor changes in viral burden, which suggests that a few protective virus-specific CD8^+^ T cells are sufficient for influenza virus clearance in the lungs.

Helminth infection induces type 2 immune responses characterized by upregulation of specific cytokines including IL-4, IL-5, IL-9, and IL-13.^24^ These cytokines polarize different immune cells such as CD4^+^ T helper 2 (T_H_2) cells and alternatively activated macrophages (AAMs) or M2 macrophages.^24^ Although CD8^+^ T cells are not directly involved in anti-helminth immunity, type 2 cytokines elicited during helminth infection can cause bystander activation of naïve CD8^+^ T cells. These activated CD44^+^CD8^+^ T cells resemble virtual memory T cells (TVM cells) that are implicated in protection against viral infections.^25,26^ In a recent study, TVM-like CD8^+^ T cells induced by systemic administration of *Schistosoma mansoni (S. mansoni) eggs* were shown to boost antiviral CD8^+^ T cell responses and protect mice against intranasal murine gammaherpesvirus (MHV)-68 infection.^27^ Similarly, coinfection with *S. mansoni* cercariae protected mice against the influenza virus strain PR8 and the paramyxovirus, the pneumonia virus in mice (PVM), a model of human respiratory syncytial virus (RSV).^28^ Although *S. mansoni*-induced mucus production in lung airways was implicated in conferring nonspecific protection to respiratory virus infection, induction of TVM-like CD8^+^ T cells might have an antiviral role against these respiratory viruses, which warrants further evaluation. Moreover, the accumulation of TVM-like CD8^+^ T cells is common during other helminth infections, including *H. polygyrus*, although whether they contribute to the protective effects seen during *H. polygyrus* and influenza virus coinfection remains unexplored. Moreover, during instances where helminth coinfection results in the systemic dissemination of gut commensal bacteria, TVM-CD8^+^ T cells might provide protection against systemic bacterial infection.^29,30^

In addition to type 2 immune responses, helminths induce an anti-inflammatory or regulatory response characterized by induction of Foxp3^+^ regulatory CD4^+^ T cells (Tregs) that suppress inflammation via production of cytokines such as IL-10, IL-35 and TGF-β.^31^ The induction of Tregs is thought to aid in the persistence of some helminth parasites in the host by impeding protective T_H_2 responses.^32,33^ However, in some settings, such as *H. polygyrus* infection, an early Treg response was reported to prevent immunopathology in mice suggesting that Tregs may be involved in multiple aspects of helminth–host interactions.^34,35^ Although helminth-induced Tregs are implicated in preventing autoimmunity and inflammation in mice,^31,36,37^ whether they suppress immunopathology caused by viral infections is not clear. It is conceivable that Treg induction due to helminth coinfection could suppress hyperinflammatory or inappropriate immune responses often associated with respiratory viral infections.^18^

Another mechanism by which helminths protect against respiratory viral infection is via induction of the type I interferon (IFN) response. Although helminths are generally not associated with the direct induction of type I IFNs, *H. polygyrus* infection was shown to upregulate type I IFNs in the lungs and protect mice against RSV.^38^ Unexpectedly, the protective effects of *H. polygyrus* were intact even in mice lacking type 2 cytokine signaling (IL-4Rα^−/-^) or adaptive immunity (RAG1^−/-^) but were lost in mice lacking type I IFN signaling (IFNAR1^−/-^). How *H. polygyrus* infection induces type I IFN in the lungs was not elucidated in this study. However, protective benefits were lost in germ-free mice, indicating a dependence on the commensal microbiota. Whether this effect occurs through systemic translocation of gut bacteria/products or via commensal bacteria/intestinal epithelial/immune cell crosstalk warrants experimental testing. It is intriguing to speculate that enteric helminths, via changes in the composition of commensal bacteria, could impact tonic IFN signaling that provides resistance to local and systemic viral infections.^39–41^ More specifically, type I IFN signaling in conventional dendritic cells was shown to support T_H_2 induction in response to *S. mansoni* egg antigen.^42^ Hence, tonic IFN signaling could enhance dendritic cell migratory activities and thereby affect immunity to viral infections. Alternatively, the type I IFN response could induce regulatory B cells that protect against immunopathology.^43^ Thus, through diverse potential mechanisms, enteric helminths can confer host resistance against respiratory viral infections and disease pathogenesis (Table 1).

**Table 1.** Murine models that examine the impact of helminth coinfection on respiratory viruses

**Respiratory** **virus**	**Helminth**	**Helminth target tissue**	**Timing of virus coinfection and** **Outcome compared to virus-only mice**	**Mechanism**	**Reference**
Influenza virus S-15	*Ascaris Suum*	Gut, liver, lungs	Varying days post helminth infection (0, 2, 4, 6, 8, 10, 12, 16). Coinfected mice had higher mortality (90%) compared to virus alone (30%) when virus infected at day 8 post helminth infection. However, mortality started decreasing when mice were infected prior to day 8 or later.	Unknown	Nayak et al., 1965^44^
Influenza virus A_2_/Japan/170	*Nippostrongylus brasiliensis*	Skin, lungs, gut	Day 0 (same day) and day 14 post helminth infection. Day 0: Coinfected mice had higher mortality (26%) compared to virus alone (6%). Lung consolidation score in coinfected mice (41%) was higher compared to virus alone (26%). Day 14: Similar mortality rates (8%) and lung consolidation scores (26%) between coinfected and virus alone infected mice.	Unknown	Wescott et al., 1966^20^
Influenza virus A_2_/Japan/170	*Heligmosomoides polygyrus bakeri*	Gut	Day 0 (same day) and day 14 post helminth infection. Lung consolidation score in coinfected mice (17%) was lower compared to virus alone (23%) when virus infected at day 14 post helminth infection but no change at day 0.	Unknown	Wescott et al., 1966^20^
Influenza virus A_2_/Japan/170	*Heligmosomoides polygyrus bakeri*	Gut	Day 14 post helminth infection. Lung consolidation score in coinfected mice (22%) was lower compared to virus alone (38%). Coinfected mice showed 100-fold lower viral titer compared to virus alone. Antibody titer against the virus was 2-fold lower in coinfected mice compared to virus alone.	Unknown	Chowaniec et al., 1972^19^
Influenza virus X31	*Trichinella spiralis*	Gut, skeletal muscle	Day 7 and day 60 post helminth infection. Day 7: Coinfected mice showed 100% weight gain by day 8 compared to virus alone (85%); similar viral titers. Day 60: No differences between virus alone and coinfected mice.	Unknown	Furze et al., 2006^22^
Influenza virus X31	*Trichinella spiralis*	Gut, skeletal muscle	Day 12 post helminth infection. Coinfected mice had 3-fold reduced virus-specific CD8^+^ T cells compared to virus-alone; similar viral titers.	Unknown	Osborne et al., 2014^23^
Influenza virus A/Puerto Rico/8/34	*Heligmosomoides polygyrus bakeri*	Gut	Day 14 post helminth infection. Coinfected mice had less than 2-fold increase in viral load compared to virus-alone; coinfected mice had 2-fold reduced virus-specific CD8^+^ T cells compared to virus-alone.	Unknown; likely due to altered immune cell trafficking.	King et al., 2014^21^
Influenza virus A/Puerto Rico/8/34 and Pneumonia virus of mice clone 15	*Schistosoma mansoni* (Omani human isolate)	Lungs, liver, blood, gut	10–12 weeks post helminth infection. Coinfected mice had lower mortality (20%) compared to virus alone (100%); coinfected mice also displayed reduced weight loss compared to virus alone.	Unknown; type I IFN-independent; likely due to TNFα- dependent goblet cell hyperplasia	Scheer et al., 2014^28^
Pneumonia virus of mice clone 15	*Schistosoma mansoni* (Omani human isolate)	Lungs, liver, blood, gut	12 weeks post helminth infection. Coinfected mice displayed reduced weight loss compared to virus alone; coinfected mice also had reduced viral load compared to virus-alone.	Unknown	Scheer et al., 2014^28^
Respiratory syncytial virus strain A2	*Heligmosomoides polygyrus bakeri*	Gut	Day 10 post helminth infection. Coinfected mice displayed reduced weight loss compared to virus alone.	Type I IFN- dependent; microbiome-dependent	McFarlane et al., 2017^38^
Murid herpesvirus 4 strain MHV-68	*Schistosoma mansoni,Nippostrongylus brasiliensis*	Lungs, liver, blood, gut	Day 22 post *S. mansoni* infection or day 6 post *N. brasiliensis* infection. Coinfected mice displayed reduced weight loss compared to virus alone; coinfected mice had 100-fold reduced viral load compared to virus-alone.	Bystander activated CD8^+^ T cells (TVM); CD8^+^ T cell intrinsic IL-4 signal	Rolot et al., 2019^27^

## Detrimental outcomes

### Enteric helminths and enteric viruses

Enteric helminths alter the GI tract tissue microenvironment including the epithelium lining the gut lumen as well as the immune cells residing in the underlying stroma.^24^ For example, helminth-induced type 2 cytokines such as IL-4 and IL-13 instruct macrophages to adopt a regulatory phenotype to promote repair of tissue damage caused by helminths.^45,46^ These IL-4-induced STAT6-dependent AAMs contrast with classically activated M1 macrophages with a pro-inflammatory phenotype. Effector molecules such as arginase-1 and RELM-α produced by AAMs regulate the synthesis of collagen constituents involved in the rebuilding of damaged tissue.^46–48^ Additionally, AAMs can suppress an unrestrained pathological immune response thereby preventing inflammation and tissue fibrosis.^49,50^ Some studies also have suggested that AAMs, together with other immune cells such as neutrophils, can kill or expel helminth larvae directly.^51–53^ These helminth-induced AAMs may be one of the cell types driving impaired host immune responses to other microbes including *Salmonella typhimurium, Citrobacter rodentium*, and *Mycobacterium tuberculosis*, and enteric viruses.^23,54–57^ In one study, the coinfection of mice with *T. spiralis* or *H. polygyrus* and murine norovirus (MNoV) resulted in enhanced viral replication in the GI tract.^23^ Specifically, Ym1, a chitinase-like molecule expressed by AAMs, impaired the proliferation of virus-specific CD8^+^ T cells, which resulted in a failure to clear the MNoV infection from the GI tract.

Another mechanism by which helminths could impact enteric viral infection is by modulating or expanding the specific cell types that viruses target. In an MNoV study, although a defect in AAM-mediated priming of virus-specific CD8^+^ T cells was implicated in enhanced viral burden, AAMs induced *in vitro* supported higher replication of MNoV.^23^ The possible skewing of AAMs by IL-4 compromises their innate antiviral functions, enabling viruses to replicate within them. Indeed, AAMs are less efficient than conventional macrophages in their ability to phagocytose antigens and kill engulfed pathogens.^45^ Helminth infection also results in the expansion of specific intestinal epithelial lineages including tuft cells and goblet cells, which have roles in the detection and clearance of luminal worms through a “weep and sweep” response.^58,59^ Coinfection of helminths and enteric viruses that have tropism for these specific cell types might result in greater numbers of susceptible target cells and increased viral infection. Notably, the MNoV strain CR6, which has tropism for tuft cells and shows higher levels of shedding following a coinfection with *T. spiralis* or during treatment with IL-4 complexes (IL-4 c).^23,60^ Similarly, coinfection with *H. polygyrus* enhanced murine astrovirus (muAstV) infection and shedding in the GI tract possibly due to an increased number of infected goblet cells, a target of muAstV.^61^ Thus, enteric helminth coinfection could be detrimental to the host by enhancing infection and transmission of some enteric viral infections (Table 2). Whether enteric helminth coinfection also enhances host susceptibility to other enteric viruses (*e.g*., rotavirus and enteroviruses) remains to be determined.

**Table 2.** Murine models that examine the impact of helminth coinfection on enteric viruses

**Virus**	**Helminth**	**Helminth Target tissue**	**Timing of virus coinfection Outcomecompared to virus-only mice**		**Mechanism**	**Reference**
Murine norovirus strain CW3 (acute) and CR6 (persistent)	*Trichinella spiralis,Heligmosomoides polygyrus bakeri*	Gut, skeletal muscle	Day 12 post helminth infection. Coinfected mice had 100-fold increased viral load compared to virus-alone; coinfected mice had 5-fold reduced virus-specific CD8^+^ T cells compared to virus-alone.		STAT6-dependent AAMs	Osborne et al., 2014^23^
Murine astrovirus	*Heligmosomoides polygyrus bakeri*	Gut	Day 12 post helminth infection. Coinfected mice had 10-fold increase in viral load compared to virus-alone.		Unknown; likely due to increase in goblet cells	Ingle et al., 2021^62^

### Enteric helminths and systemic viruses

Upon infection of the primary tissue, some viruses disseminate systemically and infect multiple organs. For example, flaviviruses such as West Nile virus (WNV), which are principally transmitted into the skin by mosquitoes, can disseminate to the brain and spinal cord after initial local replication.^63^ WNV can also spread to other organs including the GI tract, where it preferentially infects enteric neurons resulting in intestinal dysmotility.^64^ A recent study showed that *H. polygyrus* coinfection in mice exacerbated the disease caused by multiple neurotropic flaviviruses that infect the GI tract including WNV, Zika virus, and Powassan virus.^65^ Specifically, *H. polygyrus* modulated WNV infection and outcome via a tuft cell-IL-4 mediated axis, such that enteric neurons became more susceptible to WNV, resulting in disruption of the neuronal network and greater intestinal dysmotility. These phenotypes were associated with barrier function defects in the small intestine, translocation of gut commensal bacteria, systemic dissemination of bacteria, and disruption of the architecture in lymphoid tissues that resulted in a collapse of WNV-specific CD8^+^ T cell responses and elevated viral burden in the central nervous system. In this study, IL-4 alone (no *H. polygyrus* infection) was sufficient for these effects, as these phenotypes were recapitulated by IL-4 c treatment of mice prior to WNV infection.^65^ Thus, enteric helminths can enhance susceptibility to systemic viral infections that also have tropism for the GI tract. The coinfection phenotype was dependent on the expression of the receptor IL-4α on intestinal epithelial cells, suggesting that the type 2 response induced by helminths was mediated through the intestinal epithelium.^65^ In comparison, IL-4 c treatment did not alter CD8^+^ T cell responses or gut pathology after infection with the Armstrong strain of the lymphocytic choriomeningitis virus (LCMV), likely because this virus does not infect the GI tract.^65^

Helminths can affect the pathogenesis of other systemic viruses such as MHV-68 and the WE strain of LCMV.^66^ In one study, when MHV-68-infected mice were coinfected with either *S. mansoni* eggs or *H. polygyrus* larvae via oral gavage, MHV-68 reactivated from latency.^67^ IL-4 c treatment alone was not sufficient to induce MHV-68 reactivation but required a combination of IL-4 c and anti-IFN-γ suggesting that a ‘two-signal’ mechanism is needed for the reactivation of latent herpesvirus following helminth coinfection. Consistent with this idea, the coinfection with *S. mansoni* and LCMV results in reduced expression of type I IFN, namely IFN-β, and its downstream interferon stimulated genes (ISGs) in the liver.^68^ The results showed that coinfected mice had elevated viral burden, severe hepatotoxicity, and higher mortality compared to mice infected with LCMV alone. However, how helminth-induced immune responses alter IFN-β levels and which aspects of type 2 immunity was involved in this process was not addressed. Of note, the type 2 cytokines such as IL-4, IL-5, IL-10 and IL-13 were reduced in coinfected mice when compared to *S. mansoni*-infected mice suggesting a reciprocal effect of LCMV on anti-helminth immune responses.^68^ *S. mansoni* infection also impaired immune responses against systemic infection with vaccinia virus resulting in enhanced viral burden (Table 3).^69^ Thus, helminths can impair host immune responses to viral infections through local or systemic effects.

**Table 3.** Murine models that examine the impact of helminth coinfection on systemic viruses

**Virus**	**Helminth**	**Helminth target tissue**	**Timing of virus coinfection and Outcome compared to virus-only mice**		**Mechanism**	**Reference**
Recombinant Vaccinia virus	*Schistosoma mansoni*	Lungs, liver, blood, gut	7 weeks post helminth infection. Coinfected mice had increased viral load compared to virus-alone; coinfected mice showed impaired CD8^+^ T cell functionality compared to virus-alone.		Unknown	Actor et al., 1993^69^
Lymphocyticchoriomeningitis virus	*Schistosoma mansoni* (Puerto Rican strain)	Lungs, liver, blood, gut	10 weeks post helminth infection. Coinfected mice had higher mortality (80%) compared to virus alone (0%); coinfected mice had 100-fold increased viral load compared to virus-alone.		Unknown	Edwards et al., 2005^68^
Murine gammaherpesvirus-68	*Schistosoma mansoni eggs, Heligmosomoides polygyrus bakeri*, IL-4 c + anti-IFN-γ	Lungs, liver, blood, gut, systemic	Day 42 prior to helminth infection. Coinfection resulted in latent virus reactivation.		STAT6-dependent; two signal model: IL-4 and anti-IFN-γ	Reese et al., 2014^67^
Colorado tick fever virus and Eastern encephalitis virus (EEV)	*Ascaris columnaris* (*Baylisascaris procyonis*)	Gut, brain	Day 0 (same day). Coinfected mice had higher (100%) mortality compared to EEV alone (68%).		Unknown; likely due to impaired blood brain barrier	Clark et al., 1969^70^
West Nile virus (WNV), Powassan virus and Zika virus	*Heligmosomoides polygyrus bakeri*	Gut	Day 12 post helminth infection. Coinfected mice had higher mortality (75%) compared to WNV alone (15%).		STAT6 dependent; IL-4Rα expression on intestinal epithelium	Desai et al., 2021^65^

### Helminths traversing the lungs and respiratory viruses

Some human helminths such as *A. lumbricoides, A. duodenale*, and *N. americanus* have an extraintestinal phase in which the larvae migrate through different tissues such as the lungs before reaching the GI tract. As a surrogate to examine the effects of such helminths on viral infection, *Ascaris suum (A. suum)* and *Nippostrongylus brasiliensis* (*N. brasiliensis*) are used as models in mice. An earlier study found that coinfection of mice with *A. suum* and influenza virus resulted in adverse clinical outcomes.^71^ In comparison to mice infected with influenza virus alone that caused 30% mortality, coinfection resulted in 90% mortality. Moreover, the coinfected mice also died sooner than mice infected with influenza alone (5 versus 7 days) and showed pronounced dyspnea. Similar observations were made when mice were coinfected with *N. brasiliensis* and influenza virus.^20^ Coinfected mice showed higher mortality (26% vs. 6%) and greater lung consolidation scores (41% vs. 26%) compared to mice infected with the influenza virus alone. These findings suggest that a connection with helminths that traverse through the lungs and respiratory viruses can be detrimental to the host. However, the precise mechanism by which these lung-traversing helminths impact the pathogenesis of respiratory viruses and disease outcomes has not been elucidated.

### Enteric helminths and sexually transmitted viruses

In a recent study, coinfection of mice with *N. brasiliensis* was shown to exacerbate intravaginal Herpes simplex virus-2 (HSV-2) mediated epithelial ulceration in the female genital tract (FGT).^72^ *N. brasiliensis* infection alone was shown to induce recruitment of eosinophils to the FGT. However, following HSV-2 coinfection of the vaginal epithelium, local eosinophilia was enhanced, which caused damage to the virally infected vaginal epithelium. This immunopathological exacerbation occurred independently of IL-4Rα and instead depended on an IL-33/IL-5/eosinophil axis.^72^ Thus, a helminth infection that alters systemic immunity and affects the milieu of distant tissues, despite not actively colonizing those tissues, can also worsen the outcome of local viral infections (Table 4).

**Table 4.** Murine models that examine the impact of helminth coinfection on sexually transmitted viruses

**Virus**	**Helminth**	**Helminth target tissue**	**Timing of virus coinfection Outcome compared to virus-only mice**		**Mechanism**	**Reference**
Herpes simplex virus-2	*Nippostrongylus brasiliensis*	Lungs, gut	Day 7 post helminth infection. Coinfected mice had vaginal epithelial ulceration. No change in viral load compared to virus-alone.		IL-33/IL-5/eosinophil axis	Chetty et al., 2021^72^

## Determinants of coinfection outcomes

Helminth coinfection can have either a positive or negative impact on host resistance to viral infection. Although coinfections occur commonly in the real world, they are complicated to dissect as many factors can potentially influence coinfection outcome. For instance, helminth infection itself is a complex process whereby the larvae pass through different stages of their life cycle, partly inside the host and partly in the environment.^6,73,74^ Moreover, some helminths traverse through different tissues in the body and evoke a wide array of innate and adaptive immune cells.^16^ Furthermore, helminths can be expelled from the host or occupy a niche such as the GI tract and persist for long periods.^75^ Coinfection of the host with a virus that already harbors helminth parasites further adds to this complexity, as viruses may infect more than one tissue, have unique cellular tropism and can be acute, persistent, or latent. Moreover, helminth and virus can have both local and systemic effects on host immunity that can act in concert or oppose one another.^24,41^ Although the coinfection outcome is determined by the unique combination of helminth and virus, there are a few common factors that might influence whether the coinfection outcome is beneficial or detrimental. For example, what type of immune response does the helminth provoke (*i.e*., a protective or pathological immune response to virus infection)? Where does the helminth reside (*i.e*., in the same or different tissues relative to the virus)? And when does virus infection happen in relation to the life cycle of the helminth (*i.e*., during the acute or chronic stage of the helminth)? These three interrelated themes have been framed in the following sections as determinants of coinfection outcomes, namely 1) the nature of the antiviral immune response, 2) the tissue tropism of helminth and virus, and 3) the timing of viral infection in relation to the helminth life cycle.

The *nature of the antiviral immune response* is a key element in determining the outcome because helminths and viruses evoke disparate immune responses, type 2 and type 1, respectively, that can antagonize one another.^76,77^ When such contrasting immune responses are elicited in the same host during helminth-virus coinfections, the upregulation of one may suppress the other, which in turn can compromise host defenses. This is evident where enteric helminths cause defects in either innate or adaptive immune responses against viruses (Tables 2 and 3).^23,67,68^ In the setting of helminth coinfection, if antiviral immunity becomes attenuated, it could compromise control of virus infection. However, if the virus-induced immune response in infected tissues is pathological in nature and contributes to disease, then the tempered immunity due to helminth infection might be beneficial. This is evident during coinfections of enteric helminths and respiratory viruses (Table 1).^19,22^ Alternatively, bystander activation of immune cells such as CD8^+^ TVM cells could have protective roles in antiviral immunity.^27^ Thus, the nature of the antiviral immune response is a key determinant of the coinfection outcome.

**Figure 1.**
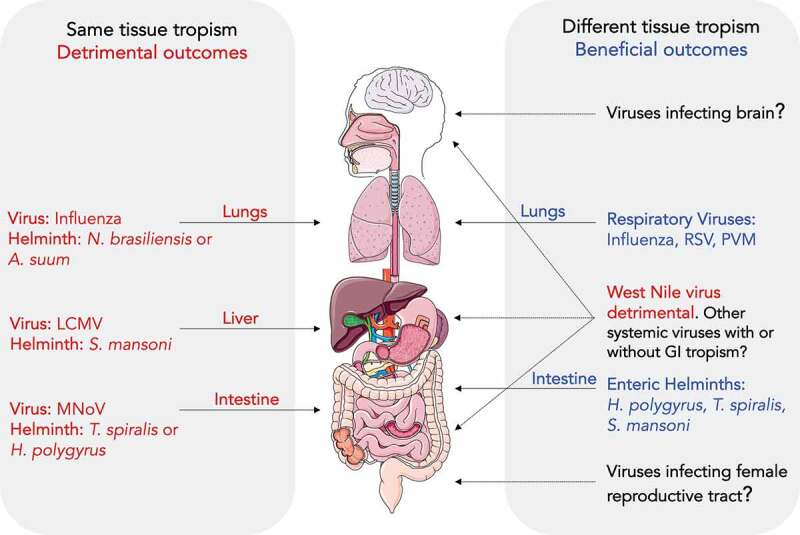
**Tissue tropism of helminths and viruses can modulate coinfection outcome**. (*Left*) Helminths and viruses that infect the same tissue can result in detrimental outcome for the host. For example, *N. brasiliensis* or *A. suum* and influenza infection of lungs; *S. mansoni*/LCMV infection of the liver; *T. spiralis*/MNoV or *H. polygyrus/*MNoV infection of the small intestine. (*Right*) Helminth and virus infection of different tissues can have beneficial effects. For example, helminths in their enteric phase such as *H. polygyrus, T. spiralis* and *S. mansoni* protect against respiratory viruses including influenza, RSV and PVM. However, *H. polygyrus* coinfection with WNV in the GI tract was detrimental to the host. The effect of enteric helminths on other systemic viruses that do not have tropism for the GI tract is unknown

Another theme emerging from helminth-virus coinfection studies is that the *tissue tropism* of the helminth and virus also affects outcome (Figure 1). This is illustrated in infection studies of the lung (Table 1). Enteric helminths alleviated respiratory viral disease likely because they occupy a different niche, gut versus lungs.^22,38^ However, influenza virus infection in the setting of lung-penetrating helminths such as *A. suum* and *N. brasiliensis*, results in worsened pulmonary disease and higher mortality rates.^20,71^ These examples support the hypothesis that the tissue compartmentalization of the helminth and the virus may determine coinfection outcome (Figure 1). Indeed, when mice were coinfected with enteric helminths and viruses targeting the GI tract such as MNoV and WNV, the intestinal viral burden was increased.^23,65^ Similarly, when mice infected with *S. mansoni* that penetrates liver tissue were coinfected with LCMV, a hepatotropic virus, local viral burden, and hepatotoxicity were enhanced.^68^ Thus, when helminth-virus coinfection occurs in the same tissue (*e.g*., lungs, liver and GI tract), it can result in detrimental outcomes.

The mechanisms underlying these detrimental effects could be diverse. Overlapping tissue tropism could compromise the induction of local immune responses against viral infections as observed in the enteric helminth and MNoV coinfection study.^23^ It could also enhance local cellular targets of viral infections as implicated in the enteric helminth and muAstV coinfection study or induce changes in viral cellular targets such as enteric neurons so that they become more susceptible to infection with WNV.^62,65^ It is also possible that helminth infection may lead to systemic enhancement of cellular targets and might increase viral replication not only at the local site but also in distant tissues, as suggested in the context of helminth-HIV coinfections where the helminth-mediated expansion of CD4^+^ T cells could promote HIV replication or transmission.^78,79^ Another possibility is that coinfection of the same tissue could exacerbate physical damage to the tissue and thereby compromise its integrity, which is likely the case during lung-penetrating helminths and respiratory virus coinfection, and *S. mansoni*/LCMV coinfection that results in damage to liver tissue.^20,68,71,80^

Beneficial effects are often seen when helminths dwell in tissues other than ones that the virus infects. This is evident in the case of the enteric helminth *H. polygyrus* and respiratory viruses such as influenza virus and RSV.^19,38^ However, *S. mansoni*, despite its capacity to transiently penetrate through the lungs, was shown to protect against intranasal infection with influenza virus strain PR8 and MHV-68.^27,28^ This can also be attributed to the timing of virus inoculation in relation to the helminth life cycle as observed in earlier studies.^20,71^ In the case of the *S. mansoni*/PR8 coinfection study, PR8 was administered during the chronic phase of *S. mansoni* (10–12 weeks later), when *S. mansoni* is no longer is present in the lungs but inhabits the portal veins and mainly affects the liver tissue. Similarly, in *S. mansoni*- and *N. brasiliensis*-mediated protection studies, MHV-68 was inoculated at time points when these helminths were no longer were present in the lung tissue.^27^ The effect of timing of virus inoculation will be discussed in more detail below. Alternatively, it is possible that the nature of antiviral immunity, such as a TVM CD8^+^ T cell response, plays a dominant role in protection despite having the same tissue niche of helminth and virus. Consistent with this, coinfection of mice with *N. brasiliensis*, a worm that does not colonize the female genital tract (FGT), was shown to exacerbate intravaginal HSV-2 induced immunopathology in the FGT.^72^ According to the hypothesis of different tissue niche, this combination of helminth–virus interactions should have been beneficial. However, *N. brasiliensis* induced alterations in systemic immunity causing a recruitment of eosinophils to FGT regions, and upon viral infection, local eosinophilia was enhanced causing damage to virally infected epithelium.^72^ The nature of antiviral immunity (immunopathological) in the helminth/HSV-2 study played a dominant role over tissue tropism and worsened outcomes. This study highlights that the helminth-virus coinfection process is complex and indicates that in some instances one particular determinant may play a dominant role in determining the coinfection outcome.

**Figure 2.**
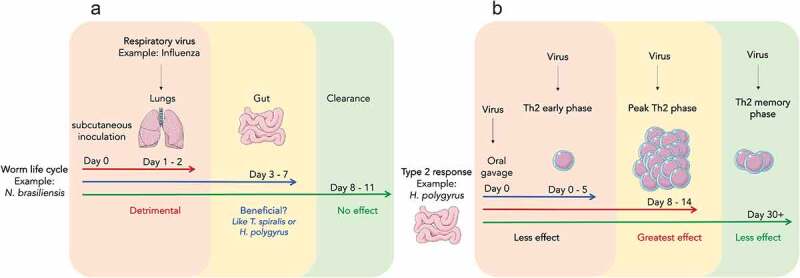
**Timing of viral infection with respect to the life cycle stage of the helminth affects coinfection outcome**. (a) Infection with influenza virus when *N. brasiliensis* is in the lungs (day 1–2) has a detrimental outcome, whereas infection with influenza at day 14, when *N. brasiliensis* has been cleared, has no effect on the host. As seen with other enteric helminths such as *T. spiralis* and *H. polygyrus*, whether infection of influenza virus during the enteric phase of *N. brasiliensis* has a beneficial effect is unknown. (b) Type 2 immune responses to the enteric helminth *H. polygyrus* peak after one week of infection, plateau, and then decline as worm burden reduces. During the peak phase, type 2 immune cells and cytokines are elevated, which can antagonize antiviral responses if virus infects during this phase. However, when virus infection occurs earlier or later, when type 2 responses are still developing or have waned, the effect on antiviral responses might be moderate, little, or none

Although not mutually exclusive with the idea of tissue tropism, the *timing of viral infection* in relation to the stage of helminth lifecycle or anti-helminth immunity may also impact coinfection outcomes (Figure 2a). Some helminths have an extraintestinal phase, whereas others are confined to the GI tract throughout their lifecycle. For example, *N. brasiliensis* and *A. suum* penetrate the lungs before establishing infection in the GI tract. If a virus infects a tissue during the time frame when helminths are present in the same tissue, it might have negative consequences in line with the *same tissue – detrimental outcome* hypothesis. However, if the virus infects a tissue that is no longer occupied by helminths or if helminths have passed through the tissue and occupy a different tissue niche, the outcome might not be detrimental. Additional evidence for this idea comes from a study showing that when mice were orally gavaged with eggs of *A. suum*, and 8 days later inoculated with the swine influenza virus, mortality was higher than in mice infected with the influenza virus alone.^80^ After oral gavage, the peak penetration period of *A. suum* in the lungs is around day 8, and thereafter the number of larvae in the lungs decline. However, when the influenza virus was inoculated either a few days before or after, rather than on day 8 after *A. suum* infection, mortality rates were reduced. Hence, the negative effect of *A. suum* and influenza virus was greatest when *A. suum* larvae were present in the lungs in high numbers. Analogous results were observed during an *N. brasiliensis* and influenza virus coinfection study (Figure 2a).^20^

The timing of viral infection also applies to coinfections with beneficial outcomes. When mice were infected with *T. spiralis*, and 7 days later infected with the influenza virus, coinfected mice showed greater weight recovery than animals infected with the influenza virus alone.^22^ However, when influenza was administered 60 days following *T. spiralis* infection, there was no change in weight recovery. This is because, following oral gavage, *T. spiralis* larvae have a peak enteric phase at day 7; the effect of *T. spiralis* on immunomodulation is likely most prominent during the enteric phase. By day 60, the larvae are encysted in skeletal muscles and may not have immunomodulatory effects. Consistent with this idea, in other studies that showed reduced lung immunopathology due to helminth-virus coinfections, the respiratory virus was administered during the time frame where helminths were in their enteric phase.^27,28,38^ Thus, timing of viral infection may be a key factor that determines the helminth-virus confection outcome.

The nature and magnitude of the type 2 immune response changes as the helminths progress through different life cycle stages. For example, early during helminth infection, innate responses are elevated, whereas adaptive T_H_2 responses occur later.^81^ The innate responses differ in terms of their role in helminth immunity during the early and late stages. For example, early during *H. polygyrus* infection, NK cells are involved in preventing immunopathology, whereas in later stages they may be dispensable or even assist T_H_2 responses.^34,82,83^ It is likely that viral coinfection occurring early during helminth infection may result in a different outcome compared to virus infection at later time points. Furthermore, during persistent *H. polygyrus* infection, the T_H_2 response peaks in the mesenteric lymph nodes around 10–14 days post-infection and then declines as the parasite burden reduces. When the virus is inoculated at the peak of the T_H_2 response, the negative effects on antiviral immunity may be greater than if the virus was inoculated at later time points (e.g., day 30+) (Figure 2b). One major factor that may influence the quality of the immune response to helminths is anti-helminth drugs or deworming treatments. Deworming causes substantial alterations in immune signatures in infected individuals, which might affect their response to subsequent heterologous pathogens.^84–86^ Consistent with this idea, in a *H. polygyrus*/WNV coinfection study, treatment of mice with an anti-helminthic prior to virus infection prevented the exacerbated mortality otherwise evident in untreated coinfected mice.^65^ However, in another setting involving the filarial helminth *Litomosoides sigmodontis*, the suppressive effects of the helminth on the quality and quantity of neutralizing antibody responses to an influenza vaccine lasted even after the helminth infection was terminated.^87^ This was due to the impact of IL-10 producing Treg cells that were sustained in the host after parasite clearance.^87^ Thus, the timing of virus infection in relation to the helminth lifecycle may or may not affect the outcome of coinfection.

## Limitations and future directions

Studies addressing the effects of helminth coinfections on viral pathogenesis are limited. Therefore, it is difficult to draw definitive conclusions with the few examples available. Moreover, the outcomes of different coinfection studies can depend on the unique nature of the helminth and the virus combination. Whether the consequences extend to other viruses that infect via similar routes or occupy similar tissue niches remains to be determined. Although the beneficial effects of enteric helminths in mitigating immunopathology caused by respiratory viral infections have been observed in the context of influenza, RSV, MHV-68 and PVM, whether it applies to emerging viruses such as SARS-CoV-2 is unknown.^88–92^ Unlike some respiratory viruses that are confined to the lungs, SARS-CoV-2 affects multiple organs including the GI tract.^93^ Since the immune response and pathogenesis of SARS-CoV-2 is systemic, the outcome of coinfection is difficult to predict.

Another limitation of current studies is the helminth dosing strategy used. In most experiments, mice were gavaged with a bolus of helminth larvae or eggs. However, in nature, helminths infections mostly occur in a recurrent fashion that can be mimicked by a ‘trickle dose’ infection design.^94,95^ Future experiments should evaluate the impact of this type of helminth infection dosing on virus coinfection. Moreover, in most studies, virus inoculation is performed at a specific time point that corresponds to the onset of patency, peak egg burden, or elevated type 2 responses (*e.g*., day 12 post with *H. polygyrus*). Since in the natural world, connections with viruses can occur at any time during the helminth lifecycle, it is important to include virus inoculations at different time points to acquire a broader picture of helminth-virus outcomes. Consistent with this idea, infection of influenza virus at varying days post-helminth infection resulted in vastly different mortality rates in coinfected mice.^71^ Host sex differences are another factor influencing the severity of helminth infection both in humans as well as in mice,^96,97^ yet coinfection studies have not been assessed for sex-based differences. Moreover, most studies examine the unidirectional effect of helminths on antiviral responses. Whether immune responses to viruses affect the helminth lifecycle remains underexplored.

Helminths alter the commensal bacteria diversity,^98^ and viral infections are affected by perturbations in commensal bacteria.^41^ Thus, it is likely that helminth-mediated changes to the microbiome can affect viral pathogenesis. However, only two studies have explored whether helminth-virus outcomes depend on changes in the microbiome.^23,38^ In one MNoV study, viral pathogenesis did not change when enteric helminth coinfections were performed in germ-free mice compared to conventionally caged mice, suggesting that helminth-induced changes in MNoV pathogenesis occur independently of the microbiome.^23^ However, for RSV, germ-free mice did not recapitulate the beneficial effects of enteric helminth coinfection in preventing RSV disease.^38^ Along with commensal bacteria, enteric helminths have cohabitated the GI tract of mammals throughout evolution. Thus, their sudden reduction in certain parts of the industrialized world due to deworming and improved sanitation is likely to affect the host–microbiota relationship.^44^ How these changes in the community structure of the microbiota affect host resistance to viral infections needs further examination. Since parasitic helminth infections in humans are often associated with malnutrition,^99^ another area of future investigation could be understanding how helminth-induced changes in host metabolism^100,101^ affect viral pathogenesis and immunity. Whether helminth-induced metabolic reprogramming compromises immune responses to viral infection warrants further exploration. Moreover, recently there has been a growing interest in understanding the effects of host microbial metabolites in influencing viral pathogenesis,^41,102,103^ and helminths, via changes in the microbiome, could regulate host responses to viral infections. Future studies of helminth–virus interactions will likely reveal additional determinants that influence coinfection outcomes as well as uncover novel mechanisms through which helminth infection affects antiviral immunity.

**Table:** 

**Outstanding Questions** In what other scenarios can enteric helminths benefit host resistance against viral infection or be detrimental? Does the *different tissue tropism – beneficial outcome* hypothesis apply to viruses that infect mucosal surfaces such as the female reproductive tract (*e.g*., Herpes simplex virus and Zika virus)? How do enteric helminths affect the pathogenesis of respiratory viruses that disseminate into multiple tissues (*e.g*., SARS-CoV-2)? Can helminth-virus coinfection studies reveal novel cross-talk between different compartments in the mammalian host such as the intestinal epithelium, enteric nervous system and systemic immunity or unravel fundamental discoveries in the gut-brain or gut-lung axis? How do enteric helminth-mediated alterations in the commensal bacteria mechanistically affect viral infections? Do helminths modulate tonic type I IFN levels that prime antiviral immunity? What do helminth-virus coinfection studies inform us about the ‘hygiene hypothesis’ and can the use of anti-helminth drugs alter host susceptibility to viral vaccines such as SARS-CoV-2 vaccines?
